# Study on ultra-high sensitivity piezoelectric effect of GaN micro/nano columns

**DOI:** 10.1186/s40580-019-0203-4

**Published:** 2019-10-22

**Authors:** Jianbo Fu, Hua Zong, Xiaodong Hu, Haixia Zhang

**Affiliations:** 1 National Key Lab of Nano/Micro Fabrication Technology, Institute of Microelectronics, Peking University, Beijing, 100871 China; 2 State Key Laboratory of Artificial Microstructure and Mesoscopic Physics, School of Physics, Peking University, Beijing, 100871 People’s Republic of China

**Keywords:** GaN micro/nano columns, Piezoelectric effect, Sensitivity, Young’s modulus, Self-organized catalytic-free growth

## Abstract

High-quality GaN micro/nano columns were prepared with self-organized catalytic-free method. Young’s modulus of GaN nanocolumns were measured under both compressive stress and tensile stress. It was found that the Young’s modulus decreases with the increasing of nanocolumn diameter due to the increase of face defect density. Furthermore, we measured the piezoelectric properties and found that there was a 1000-fold current increase under a strain of 1% with a fixed bias voltage of 10 mV. Based on the Schottky Barrier Diode model, we modified it with the effect of polarization charge, image charge and interface state to analyze the experiment results which reveals that the strong piezopolarization effect plays an important role in this phenomenon. Therefore, the GaN nanocolumns has a great prospect to be applied in high-efficiency nanogenerators and high-sensitivity nanosensors.

## Introduction

With the development of wearables and electronic skin in recent years, it is an urgent need to improve the performance of sensors and power supply devices while reducing their sizes. So that high efficiency energy transduction methods, which can convert the human body activities into electric signal, are explored widely. Of varied methods, piezoelectric effect has been studied a lot as one of the major branches of nanogenerators and nanosensors for its excellent performance. For example, ZnO nanowires have been proven to be very effective in collecting mechanical energy at the micro/nano scale, and they are also well tolerated, highly efficient, and biocompatible [1]. Earlier researches on the piezoelectric properties of ZnO nanowires were led by Wang’ group [2]. It is believed that due to the strong piezoelectric effect of ZnO, the contact potential between the nanowire and the silver gel changes when the stress is applied. Therefore, the I–V characteristics change accordingly. And they verified the theoretical expectations by atomic force microscopy (AFM) experiments. These works gained widespread attention and made ZnO nanowire a hotspot for nanogenerators [3, 4]. Subsequently, similar experiments have been done on CdS [5], GaN [6] and InN [7]. These basic studies have laid the foundation for piezoelectric nanogenerators [8] and nanosensors [9].

Benefit from its unique crystal structure, GaN low dimensional material has strong spontaneous polarization effect and piezoelectric polarization effect. Compared with ZnO materials, the advantage of higher piezoelectric coefficient [10] makes GaN more suitable for nanosensor and nanogenerator [11–13]. On the other hand, the preparation process of P-type GaN materials is more mature than that of ZnO [14–17], which makes it has a greater potential in optoelectronic integration in the future. However, due to the difficulty in the preparation of high-quality GaN nanocolumns, there is few works on the mechanical and piezoelectric properties of micro/nano columns.

In this paper, high-quality GaN micro/nano columns were grown, and their mechanical properties and piezoelectric properties were studied. It is found that there is an ultra-high sensitivity piezoelectric effect in these GaN micro/nano columns. That makes them have a great application prospect in piezoelectric nanosensors and nanogenerators [18, 19].

## Self-organized catalytic-free growth of GaN nanocolumns

**Fig. 1:**
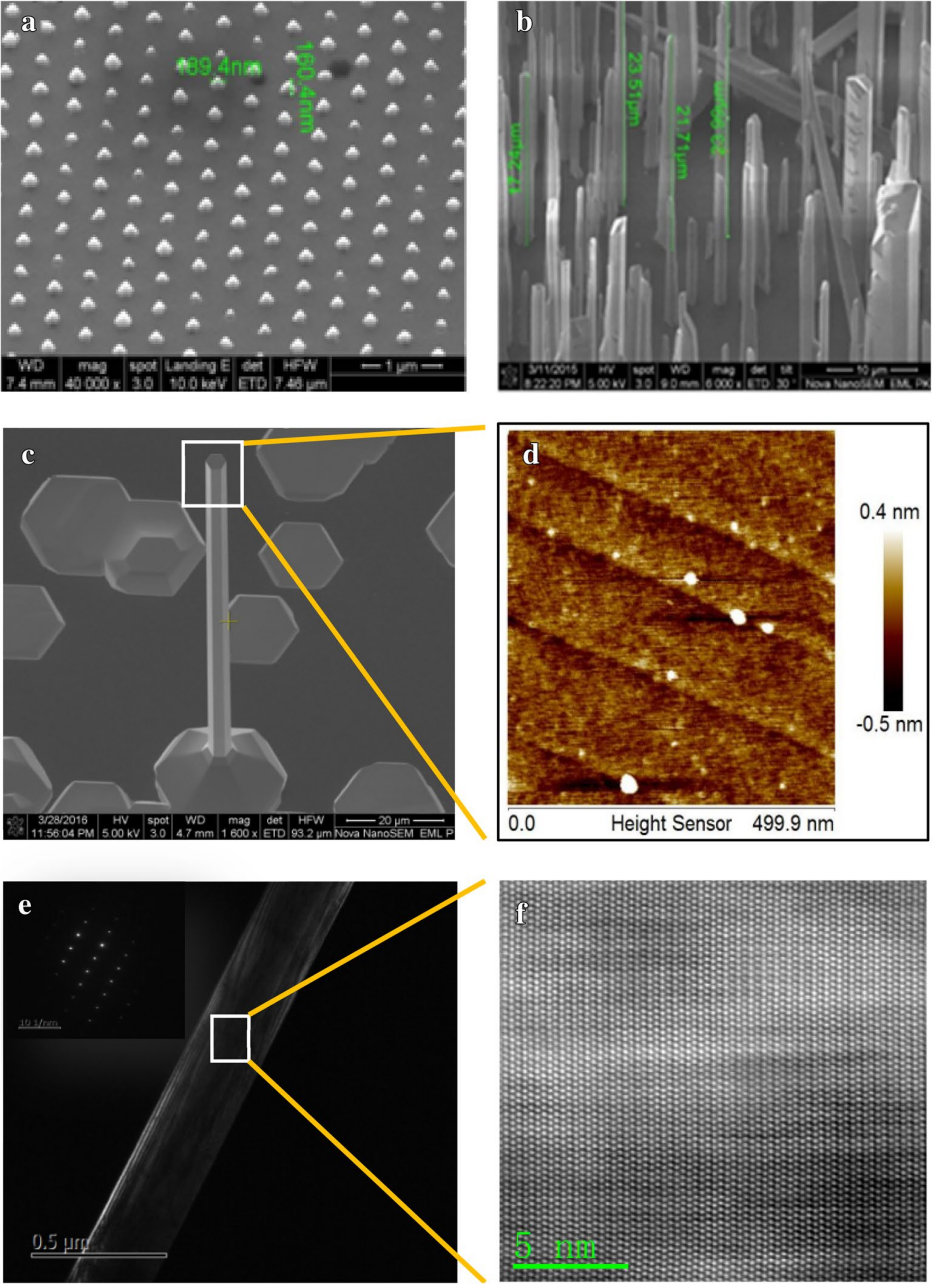
Typical scanning electron microscope (SEM) images of GaN nanocolumns grown by **a** selection growth method and **b** self-organized method, **c** SEM image of the self-organized catalytic-free growth GaN nanocolumn, **d** AFM image of the top of the GaN nanocolumn, **e** transmission electron microscope (TEM) image and electron diffraction pattern, **f** high resolution TEM atomic image

In a variety of GaN nanocolumn growth methods, self-organized growth has the advantages of simplicity and convenience than selection growth method for there is no need to prepare complex substrates [20, 21], which makes it a very important method for scientific research and industry. The typical morphology of GaN nanocolumns grown with selection growth method and self-organized method can be seen in Fig. 1a, b, respectively. On the other hand, it is desirable for catalytic-free growth of GaN nanocolumn because that the catalyst can contaminate materials [22–25].

We obtained high quality GaN micro/nano columns by self-organized catalytic-free growth method. It can be seen from Fig. 1c, the top of the nanocolumn presents a regular hexagonal shape with sharp edges. AFM image in Fig. 1d shows monoatomic steps on the top surface, which indicates that the micro/nano column was grown well. TEM images in Fig. 1e, f characterize the crystal orientation along the column is (0001) and the crystal is of high quality.

## Results and discussion

### Mechanical properties of GaN micro/nano columns

Young’s modulus is a measure of the stiffness for an elastomer which is defined as the ratio between stress and strain in the range applicable to Hooke’s law. Meanwhile, it is a tensor. The stress is expressed as force *F* divided by area *S*. The strain is expressed as Length change *ΔL* divided by length *L*. So, the Young’s modulus *E* is expressed as:

1 $$ E = \left( {F\,\cdot\,L} \right)/\left( {S\cdot\Delta L} \right) $$

where *F*, *S*, *ΔL*, *L* represent the force applied, the cross-section area, the length variation under the applied force and the total length, respectively. In this paper, we only studied the elastic Young’s modulus of GaN nanocolumns along the (0001) direction to simplify the phenomenon.

The mechanical tests in this paper were performed on Hysitron’s Picoindenter micro/nano mechanics test system. It has a three-plate capacitive sensor to measure the loading force and displacement accurately. The sample was fixed on Specimen, which converts the displacement signal into an electrical signal through a capacitive sensor and obtains the mechanical parameters accurately with feedback system.

**Fig. 2:**
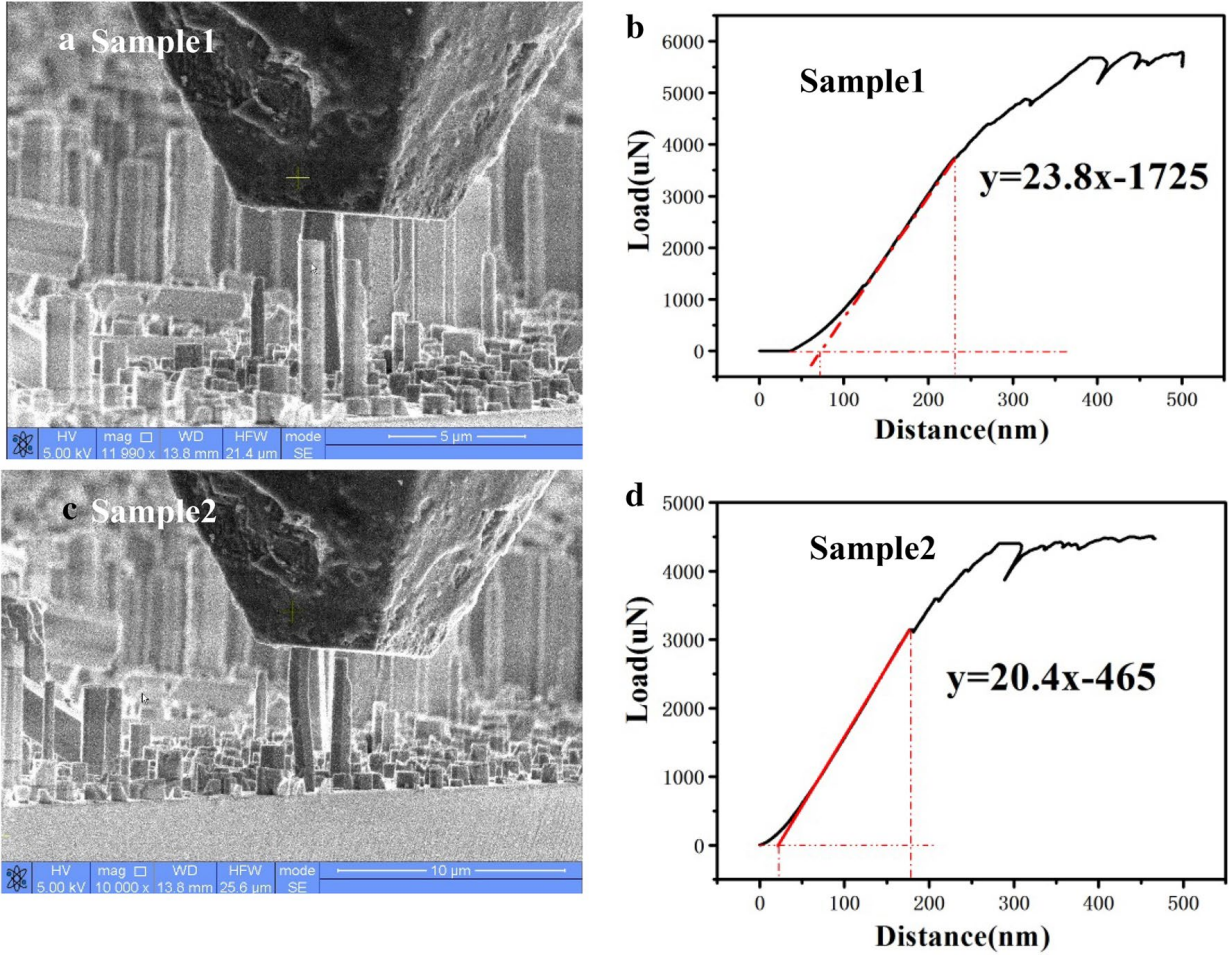
Compressive stress test by Hysitron sample stage. **a**, **b** are the test process and result of sample 1, **c**, **d** are the test process and result of sample 2

We marked the first tested GaN nanocolumn as sample 1, which has a diameter of 1080 nm and a length of 8.0 μm. The system measured the feedback pressure when the quadruple indenter pressed against the nanocolumn. As the pressure gradually increased, the nanocolumn bended as shown in Fig. 2a. As the pressure further increased, the nanocolumn broke. From Fig. 2b, it can be seen that the middle section of the force curve is linear, which represents the plastic deformation of the nanocolumn. The first nonlinear curve is due to the contact error of the indenter, which can be subtract by linear fitting. The nonlinear curve of the last segment represents the crack inside the nanocolumn. Figure 2c, d record the stress versus strain for another nanocolumn marked as sample 2, which has a diameter of 823 nm and a length of 6.6 μm. As it can be seen from the Fig. 2d, the trend of the curve is basically the same as that of Fig. 2b. The cross-section area of hexagonal column can be calculated by:

2 $$ {\text{S}} = \frac{3\surd 3}{8}D^{2} $$

where D is the diagonal of hexagonal column, which is the diameter. In order to subtract the contact error, we linear fitted the plastic deformation region of force curve in Fig. 2b, d and get the slope rate to replace F/∆L to calculate Young’s modulus. The slope rates in Fig. 2b, d are 23,800 N/m and 20,400 N/m, respectively. According to Eq. (1) and (2), there is:

3 $$ {\text{E}} = \frac{8\surd 3}{9}\frac{F}{{D^{2} }}\frac{L}{\Delta L} = \frac{8\surd 3}{9}\frac{L}{{D^{2} }} \cdot Slope\;rate $$

With the data above, the calculated compression moduli according to Eq. (3) are about 250 GPa for sample 1 and 306 GPa for sample 2. These Yong’s moduli are lower than that of GaN bulk material (300 GPa). Based on these experimental results, we found that the larger the diameter of the nanocolumn, the smaller the compression modulus. This may be because that it has more defects when the diameter is increased. However, these compression moduli have large errors because the pressures were not along the C axis when the nanocolumns bended under force. Therefore, in order to obtain more accurate experimental results and verify the inference, tensile stress test should be done.

**Fig. 3:**
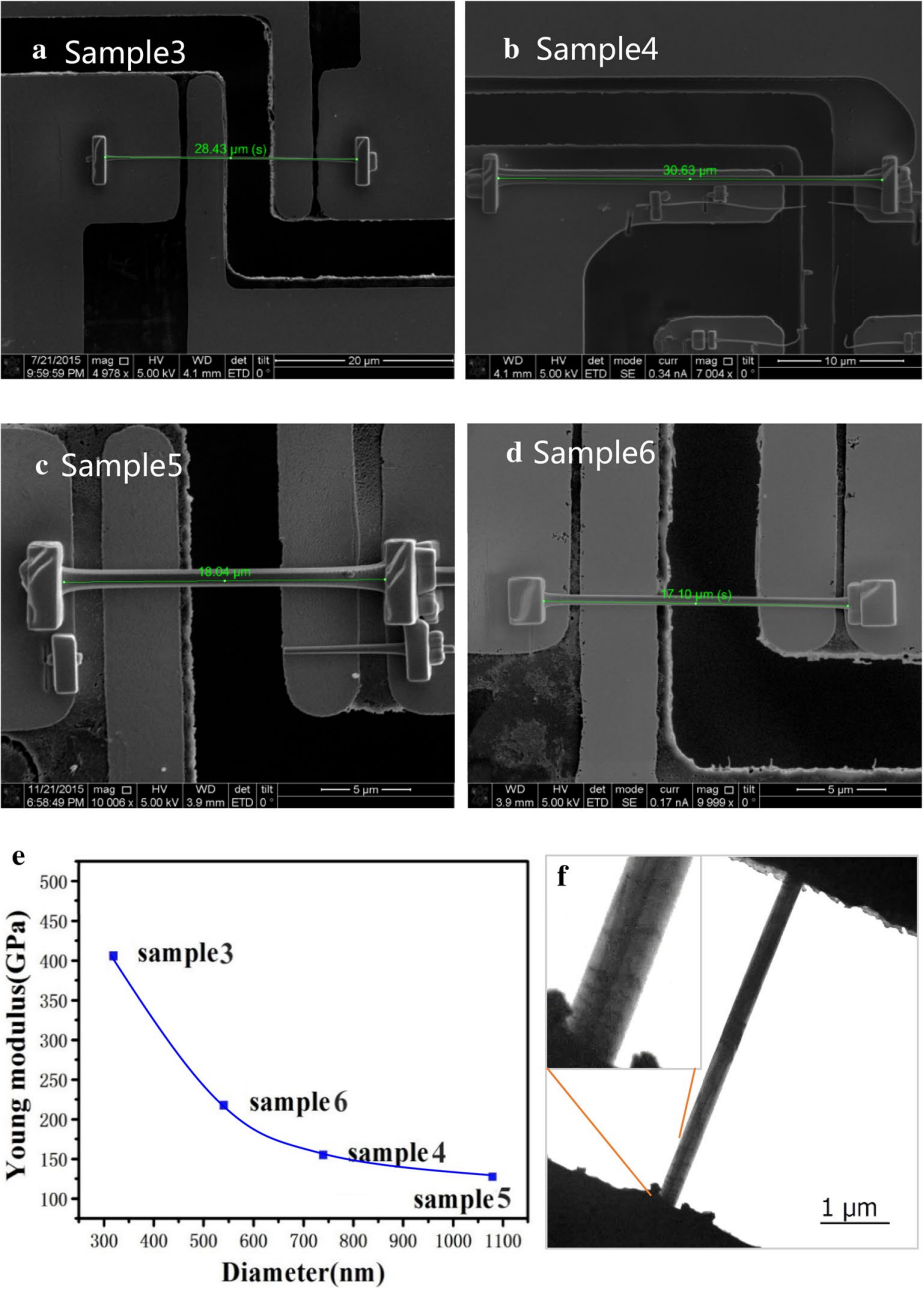
**a**–**d** Tensile test nanocolumn samples successfully prepared by Helios 600FIB. **e** The Young’s modulus obtained for the four samples, and **f** the TEM image of Sample 3, which shows the surface defects

In the tensile stress test, nanocolumns of appropriate length and diameter were selected by Helios 600FIB. The nanocolumns were stress free attached to a Push to Pull (PTP) sample stage. The free ends were soldered to the sample stage by deposited Pt. The test sample morphology is shown in Fig. 3a–d. A special probe was used to push the left half of the sample stage and then the gap between the left and right parts of the sample stage increased. Since the nanocolumn was soldered, it was subjected to the uniaxial tensile stress along C-axis. The mechanical results are shown in Fig. 3e. It can be seen that the Young’s modulus decreases as the diameter increased, which is according with the result of compressive stress test above. This result deviates from the prediction of Espinosa that the Young’s modulus of GaN nanocolumns will reach the value of bulk GaN material when the diameter exceeds 300 nm [26]. In our experiments, the Young’s modulus has become half of the Young’s modulus (300 GPa) of the GaN bulk material when the diameter is larger than 500 nm. The deterioration in the mechanical properties of the nanocolumns might result from the increase of defects density. Among the many kinds of defects, we think the surface defects contribute the most to the decrease of Young’s modulus, which can be clearly seen in Fig. 3f.

To our knowledge, it is very difficult to eliminate all defects in GaN nanocolumn. In order to estimate the effect of defect, we can make some approximations. First, we fixed GaN nanocolumn diameter as 1 μm. And we only consider the situation that face defect perpendicular to the column to simplify the calculation. According to Ref. [27], there is a relationship as below [27]:

4 $$ \frac{1}{E} = \frac{{V_{cystal} }}{{E_{cystal} }} + \frac{{V_{defect} }}{{E_{defect} }} $$

where $E_{cystal}$, $E_{defect}$, $V_{cystal}$, $V_{defect}$ are the Yong’s modulus of single crystal and defect part.$V_{cystal}$, $V_{defect}$ are their volume ratio, respectively. Based on Ref. [28], we roughly approximate $E_{defect} = 10$ GPa [28]. It was found that the the Young’s modulus will decrease from the bulk material 300 GPa to 190 GPa and 139 GPa with surface defects affected volume ratio of 2% and 4%, respectively. Therefore, based on these experiment results and simulating results, we can draw a conclusion that the Young’s modulus of GaN micro/nano columns will be decreased by defects.

### Piezoelectric properties of GaN micro/nano columns

Unlike to the mechanical properties, the electrical properties of nanocolumns are more sensitive to many experimental conditions. Therefore, we must control the soldering current, chamber vacuum, electron beam current, voltage scanning step size and rate more subtle to obtain valid electrical results. Not described in detail here.

**Fig. 4:**
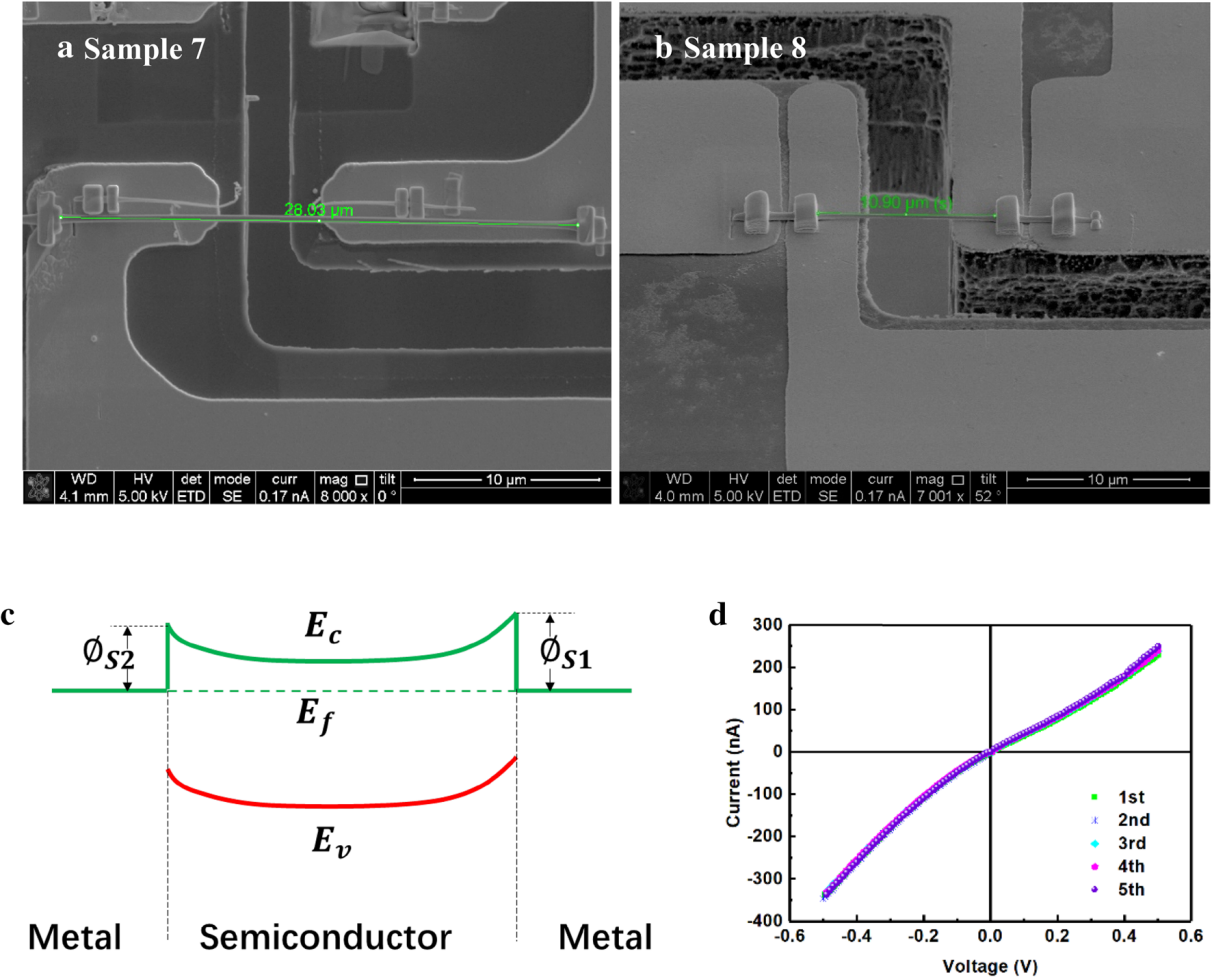
**a** Sample 7 and **b** sample 8 were prepared for piezoelectric test. **c** Schematic of the energy band diagram of the two electrodes of a GaN nanocolumn, **d** the repeat electrical curves in sample 8

Now, we analyze the electrical model based on the electrical data. Generally, Schottky barrier will be formed between the Pt and GaN contact due to the difference in work function [29]. It’s usually called a Schottky Barrier Diode (SBD). As we know, a positive bias turns the SBD on, and a reverse bias turns it off. In this experiment, Schottky barriers were formed at both ends of the nanocolumn, the schematic energy band diagram is shown in Fig. 4c. Therefore, no matter what the current direction is, the electrons will pass through a forward biased SBD and a reverse biased SBD. So that the electrical properties will be dominated by reverse biased SBD for it takes most of the voltage drop. The basic model of a single SBD is described by Eq. (5) [30].

5 $$ I = I_{s} \left[ {exp\left( {\frac{qv}{nkT}} \right) - 1} \right] $$

6 $$ I_{s} = SA^{*} T^{2} \;exp\left( { - \frac{{q\phi_{s} }}{kT}} \right) $$

where *S* is the electrode contact area, A* is effective Richardson constant, *T* is temperature, *k* is the Boltzmann constant, *q* is the charge quantity, *n* is ideal factor, and *∅*_*s*_ is Schottky barrier height. When the reverse voltage *V* is applied, the exponential term tends to zero, which is negligible, so the current *I* is approximate to − *I*_*s*_. Therefore, for the model shown in Fig. 4c, the reverse bias curve of − *I*_*s*_ should be obtained under both forward and reverse biased voltages. However, after many repeat electrical measurements, as shown in Fig. 4d, it is found that the electrical curve is double J-shape curve instead of double cut-off shape. This indicates that a simple Schottky barrier model is not suitable for these GaN nanocolumn samples. Obviously, some corrections based on the original model are needed.

**Fig. 5:**
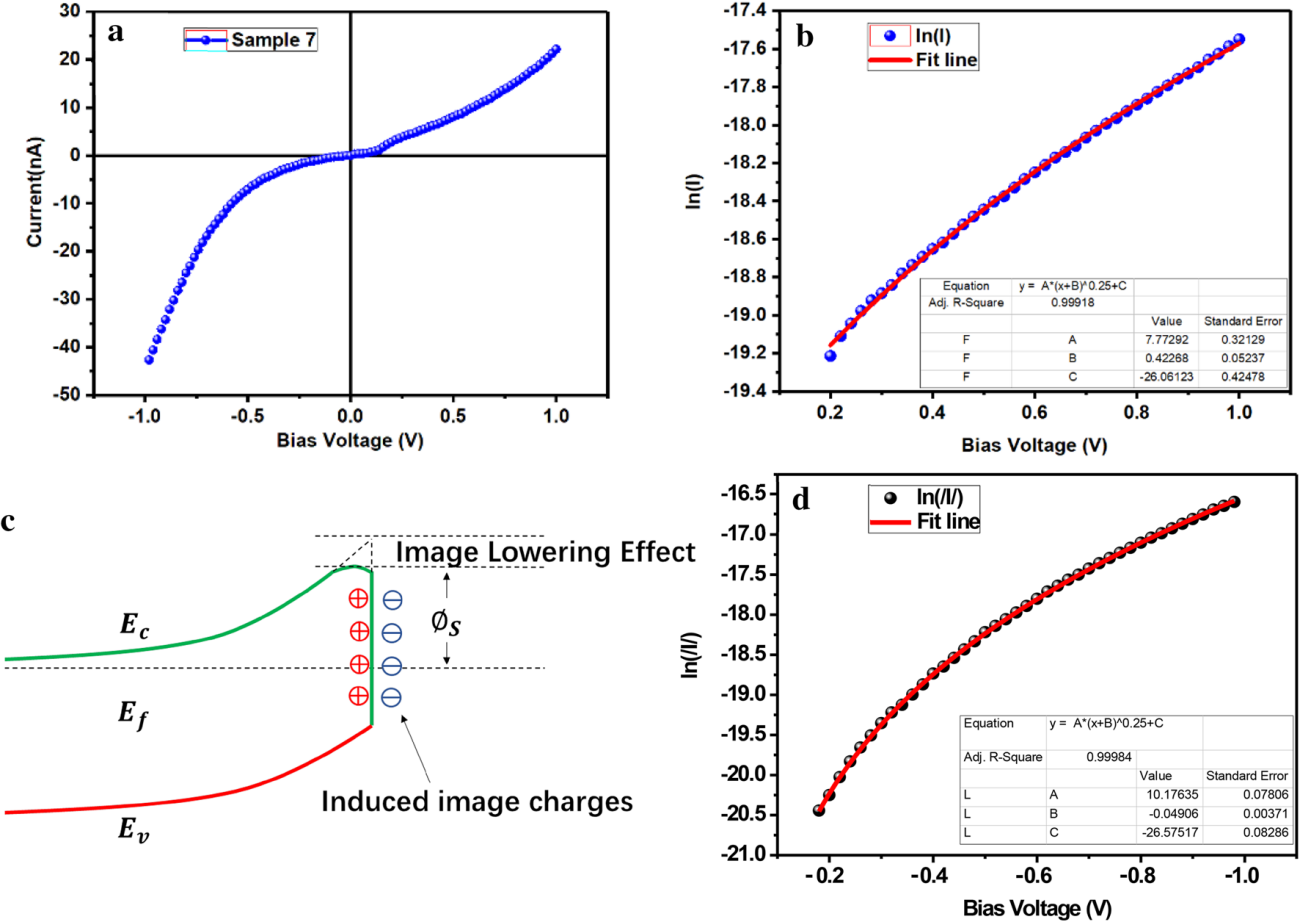
**a** Electrical characteristics of sample 7, fitting the **b** positive part and **d** negative part of electrical curve of sample 7 with Eq. (10), **c** Energy band diagram considering the image force

The double J-shape curve in Fig. 5a is taken as an example to discuss the modification of the origin model. Due to the complex interface conditions between the deposited Pt electrode and the GaN nanocolumn, the Schottky barriers have different heights as schematic in Fig. 4c. Therefore, the double J-shape curve is asymmetrical [31]. As we know that the GaN nanocolumn has strong self-polarization effect, so image lowering effect should be considered first to modify the model. At the metal and semiconductor contact interface, some charges accumulate at the semiconductor side because of polarization or Fermi level difference. As a result, some opposite charges are induced at the metal side due to the Coulomb force of the accumulated charges in semiconductor side. These induced charges will lower the Schottky barrier at the interface, which is known as Image Lowering Effect. The effect of the image force on the height of the Schottky barrier is schematically shown in Fig. 5c. Basic on this analysis, we derive a new mathematical model, which is corrected by the image force [32].

7 $$ I_{s} = SA^{*} T^{2} \;exp\left( { - \frac{{q\phi_{s} }}{kT}} \right)\;exp\left( {\frac{{\sqrt[4]{{q^{7} N_{D} \left( { - V + \varphi_{bi} - kT/q} \right)/\left( {8\pi^{2} \xi_{s}^{3} } \right)}}}}{kT}} \right) $$

8 $$ ln\;I_{s} = ln(SA^{*} T^{2} ) - \frac{{q\phi_{s} }}{kT} + \frac{{\sqrt[4]{{q^{7} N_{D} \left( { - V + \varphi_{bi} - kT/q} \right)/\left( {8\pi^{2} \xi_{s}^{3} } \right)}}}}{kT} $$

9 $$ = ln(SA^{*} T^{2} ) - \frac{{q\phi_{s} }}{kT} + \sqrt[4]{{\frac{{q^{7} N_{D} }}{{8\pi^{2} \xi_{s}^{3} k^{4} T^{4} }}}}\sqrt[4]{{\left( { - V + \varphi_{bi} - kT/q} \right)}} $$

10 $$ ln\;I_{s} = A\sqrt[4]{{\left( {x + B} \right)}} + C $$

where $$ {\text{A}} = \sqrt[4]{{\frac{{q^{7} N_{D} }}{{8\pi^{2} \xi_{s}^{3} k^{4} T^{4} }}}} $$, $$ {\text{B}} = \varphi_{bi} - kT/q $$, $$ {\text{C}} = ln(SA^{*} T^{2} ) - \frac{{q\phi_{s} }}{kT} $$, $$ x = - V. $$

Here $N_{D}$ is the donor doping concentration, $\varphi_{bi}$ is the construction potential in junction area, $\xi_{s}$ is the dielectric constant of GaN. Based on the origin model, an exponential term is added to describe the effect of the image force. By the derivation of Eqs. (7) and (9), an abstract form of Eq. (10) is obtained. Qualitatively, since the image force item related to the applied voltage *V*, it explains the appearance of double J-shape curve. In order to verify this new model, we fit the positive part and negative part of the electrical curve in Fig. 5a with a formula form of Eq. (10). The fitting results are shown in Fig. 5b, d. It can be seen that the new model fits the electrical characteristics of sample 7 quite well.

Next, we discuss the electrical properties under stress. When the nanocolumn is subjected to tensile stress, due to the piezoelectric effect of GaN, the two ends of the nanocolumn spontaneously generate charges. These charges will affect the Schottky barrier, and further affect the electrical properties.

**Fig. 6:**
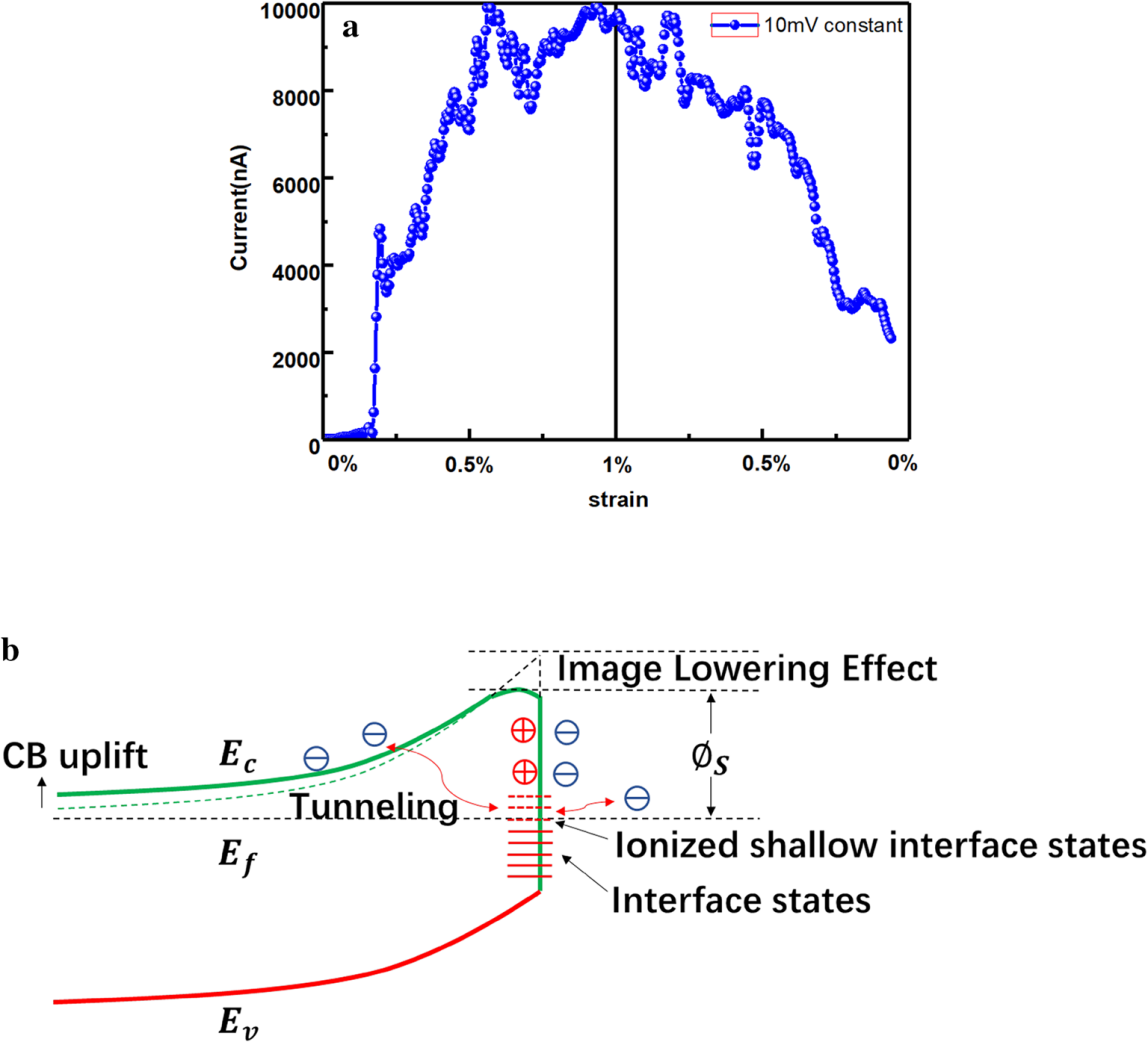
**a** Current vs. strain under constant bias of 10 mV on sample 7, **b** the schematic energy band model with considering the interface states

Based on sample 7, the effects of stress on electrical properties have been studied. During the test, we obtain the current under a constant bias voltage of 10 mV when the nanocolumn was uniaxially stretched. The experimental result is shown in Fig. 6a. We have done the same experiment on sample 8 and obtained similar result. It can be seen from Fig. 6a that the current increases from the initial 10 nA to the peak of 10 μA. The current changed nearly 1000 times while the strain was 1% only, this indicates that the GaN nanocolumn has a potential to be ultra-high-sensitivity sensors.

To analyze the electric properties under stress, important consequence of the stress should be considered. We think there are four factors are important here. They are polarization charges, image charges, energy band uplift and interface states. We take some simulation and calculation to explain the experiment results.

We analyze the effect of polarization charges first. If the eigen doping density of the GaN nanocolumn is assumed to be 1 × 10^17^/cm^3^, the surface polarization charge density at both ends of the nanocolumn is about 4.56 × 10^16^e/m^2^ when the nanocolumn strain is 1% according to the piezoelectric equation. We simulated the energy band with commercial Crosslight by setting the calculated polarization charge density as a parameter in the software. It is found that the Schottky barrier height will be reduced about 30 meV, which will increase the current by 3.5 times.

And then we consider the effect of image charges. After careful analysis, it was found that the effect of image charges induced by polarization charges is significant. When the GaN nanocolumn strain reaches 1%, the image charges will lower the Schottky barrier about 70 meV, which can make the current increases about 12 times.

Next, we discuss the energy band uplift and the interface states [33] together. Based on calculations, we found that the energy band will be uplift about 30 meV when the strain reaches 1%. This effect also can make the current increase about 3.5 times. As we know, the interface defects will form interface states in the energy band. These interface states usually distribute between the valence band and conduct band. Under the tensile stress, these interface states might be uplift to be shallow interface states, which will be easily ionized due to the strong piezoelectric polarization effect. These ionized shallow interface states can be the springboard for electrons to tunneling through the Schottky barrier [34–36]. Figure 6b explains this step tunneling effect schematically. With the stress increasing, more and more interface states are ionized due to the enhance of band uplift effect and polarization effect. This stepped tunneling effect can be equivalent to a lowering effect to the Schottky barrier. As the consequence, the current dramatically increases with the increasing of stress. We assume an interface states density of 1 × 10^14^/(eVcm^2^), the current increased by stepped tunneling effect equals about 45 meV Schottky barrier lowering effect at the strain of 1% along C-axis. This will make the current increase about 6.5 times.

Based on the discussion above, it can be seen that the current can be increased by more than 1000 times when the strain reaches 1% under these four effects. And also, it was found that the piezoelectric effect plays a key role in our experiment because all four important effects are caused by it.

## Conclusion

In-situ compression modulus tests were performed along C-axis of GaN nanocolumns in the SEM using Hysitron’s mechanical sample stage. Uniaxial tensile stress modulus measurements were done with Hysitron TI950 MEMS devices. It was found that the Young’s modulus decreases with the increasing of nanocolumn diameter, which is deviate to the prediction of Espinosa’s group. This may be because of the increased density of the surface defects, which has been supported by simulating results.

According to the measured electrical data, a phenomenon of 1000-fold current increase at 1% strain was observed. A model based on the Schottky barrier, and also considered polarization charge, image charge and interface states, was introduced to explain the experiment data. These research shows that the strong piezoelectric effect plays an important role in this current increase phenomenon. It can be hypothetical in a GaN nanogenerator device that the strong piezoelectric effect plays a role of engine and the Schottky barrier at the contact interface play a role of amplifier which can strongly enhanced the current in the circuit. This kind of device has prospect applications in high-efficiency energy harvesting and high-sensitivity detection.

## Data Availability

The datasets used and/or analysed during the current study are available from the corresponding author on reasonable request.

## Abbreviations

MESFET: metal field effect transistors
HFET: heterojunction field effect transistors
UV: ultraviolet
AFM: atomic force microscopy
SEM: scanning electron microscope
TEM: transmission electron microscope
SBD: Schottky Barrier Diode
